# Chinese translation and validation of a parental feeding style questionnaire for parents of Hong Kong preschoolers

**DOI:** 10.1186/1471-2458-14-1194

**Published:** 2014-11-21

**Authors:** Wilson Tam, Vera Keung, Albert Lee, Kenneth Lo, Calvin Cheung

**Affiliations:** Alice Lee Centre for Nursing Studies, Yong Loo Lin School of Medicine, National University of Singapore, Singapore, Singapore; Jockey Club School of Public Health and Primary Care, The Chinese University of Hong Kong, Shatin, Hong Kong; Center for Health Education and Promotion, Jockey Club School of Public Health and Primary Care, The Chinese University of Hong Kong, Shatin, Hong Kong

**Keywords:** Validation, Parental feeding style questionnaire, Childhood obesity

## Abstract

**Background:**

Childhood obesity is a major public health issue in many countries, including China. The importance of parenting relative to the healthy development of children requires the development of instruments for assessing parental influence on child dietary pattern. This study aimed to confirm the internal reliability and validity of a self-report measure on parental feeding styles, including emotional feeding, instrumental feeding, prompting or encouragement to eat, and control over eating.

**Methods:**

A 27-item parental feeding style questionnaire (PFSQ) was translated into Chinese and then translated back into English to verify consistency. The questionnaire was then used to conduct a cross-sectional survey on the parents of Hong Kong preschoolers. The internal reliability and validity of the questionnaire were examined by Cronbach’s alpha and exploratory factor analysis, respectively.

**Results:**

4,553 completed questionnaires were received. Cronbach’s alpha of subscales ranged from 0.63 to 0.81, and the overall reliability was good (alpha = 0.75). The factor structure of this questionnaire was similar to that of the original and Turkish versions. One-factor structure was identified for emotional feeding, instrumental feeding (four items), and prompting or encouragement to eat, whereas a two-factor structure was revealed for control over eating.

**Conclusion:**

The Chinese version of the PFSQ has good reliability and validity in assessing parental feeding styles in Hong Kong. Researchers can use this instrument to improve their understanding on how parental feeding styles may affect the dietary patterns and ultimately the weight statuses of children among Chinese-speaking populations across different countries.

## Background

Early life is a crucial period for human development. Various risk factors that are exposed during early life are attributed to the global burden of non-communicable diseases [1]. A recent review reported that childhood obesity, which is one of the early life risk factors, predicts adult obesity; [2] weight gain after mid-childhood can result in greater adverse effect on later risk factors of cardiovascular diseases [3]. Childhood obesity can also adversely affect almost all organ systems, bringing serious physiological and psychological consequences [4].

Childhood obesity has become one of the major public health issues in China. The prevalence of obesity among Chinese children has increased dramatically from 4.1% in 2000 to 8.1% in 2010 [5]. A similar phenomenon has been observed in Hong Kong; according to the Department of Health, Hong Kong Special Administrative Region Government, the obesity rate among primary school students rose from 16.4% in 1997–1998 to 21.3% in 2007–2008 [6]. Obesity, specifically central obesity, increases the risk of other health problems, such as diabetes, heart disease, and hypertension, and the costs associated with obesity can account for 10% to 20% of Hong Kong’s total public expenditure on health every year [7]. Therefore, various obesity-related chronic diseases in later stages of life can be prevented by teaching healthier eating habits to children.

To ensure the healthy development of children, parents do have vital roles. The limited autonomy and dependence on adult caretakers cause the dietary patterns of children to be affected by the foods provided and social environment created by caretakers [8]. Parental feeding style, which refers to the specific parental techniques or behavior to influence the food intake of children, [9] can be a major factor in explaining how parents influence the children’s diet and even their body weight. Studies have shown that parental feeding style may be associated with children’s fruit and vegetable consumption and weight status [10]. Parental feeding style has also been associated with childhood fruit and vegetable intake, [11] high fat and/or sugar intake, [12] dairy intake, [13] and weight [14].

The most updated systematic review has identified 71 instruments developed for measuring parenting feeding style. These feeding practices can be categorized into physical, social, cultural and political aspects [15]. Since parental feeding is a potential modifier on the association between general parenting and child weight, assessment tools of feeding practice should have close conceptual alliance with parenting style. Unfortunately, limited instruments have fulfilled this criteria except Caregiver’s Feeding Style Questionnaire (CFSQ), Infant Feeding Style Questionnaire (IFSQ), Child Feeding Questionnaire (CFQ) and Parental Feeding Style Questionnaire (PFSQ) [16]. Parental control over eating is the most common dimension of four instruments (used by CFSQ, IFSQ and PFSQ). However, only PFSQ assesses instrumental, emotional and encouragement feeding, which reflects consistency, autonomy-encouraging and over-protectiveness in general parenting respectively [16]. Despite being independent of child weight, [17] instrumental and emotional feeding is associated with greater child food responsiveness [18].

The PFSQ has been widely used [19–22] and has been translated and validated in Turkish [23]. Nevertheless, a validated instrument remains unavailable in Chinese. Previous studies used self-developed questions to measure parental feeding style [24, 25]. Therefore, the current study aimed to translate a validated parental feeding style questionnaire (PFSQ) [26] into Chinese and examine the internal reliability and validity of this tool among parents of Hong Kong preschoolers.

## Methods

### Subjects

Eligible participants included parents of kindergarten students in Hong Kong.

### Instrument

The PFSQ developed by Wardle et al. [26] was used in the current study. This questionnaire contained 27 items for four scales, namely, emotional feeding (five items), instrumental feeding (four items), prompting/encouragement to eat (eight items), and control over eating (ten items). Five items in control over eating were reversed questions. Respondents were asked to choose from a five-point Likert-scale for each item, ranged from never (1 point) to always (5 points). A higher average score on each scale implied higher tendency for parents to adopt particular style. As measured by Cronbach’s alpha, the internal reliability of emotional feeding, instrumental feeding, prompting or encouragement to eat, and control over eating were 0.83, 0.67, 0.74, and 0.81, respectively.

### Demographic and other variables

Demographic variables of the children included gender, grade, date and place of birth, and number of siblings, whereas those of the parents included working status and educational level.

### Translation

A translation-back-translation procedure [27] was applied to the PFSQ to obtain a Chinese version. First, the original English version was translated into Chinese by one investigator. The Chinese version was then translated back into English by another investigator. The English translation was compared with the original PFSQ, and minor discrepancies were revised based on consensus. The completed questionnaire has been pre-tested in a convenience sample of 10 parents from a kindergarten.

### Procedure

A cross-sectional survey was conducted among Hong Kong preschoolers in December 2010. Letters of invitation were sent to 100 Hong Kong kindergartens, which had previously participated in a health promotion school project (http://www.cuhk.edu.hk/med/hep/). Twenty-seven kindergartens agreed to participate in the study. Self-administered questionnaires with a consent form on the first page were distributed among the parents or guardians from the participating kindergartens. Parents were allowed to refuse to join the study by returning a signed refusal form and the blank questionnaire. The parents were also advised not to discuss the study with their children while filling the questionnaires. Completed questionnaires were collected from the kindergartens within two weeks after distribution. Ethical approval was granted by the Survey and Behavioral Ethics Committee of the Chinese University of Hong Kong.

### Data analysis

Descriptive statistics were used to summarize the demographic variables. The five reversed items were re-coded before computing for the average of the four scales. Internal reliability was assessed by Cronbach’s alpha for the four scales. Exploratory factor analysis, using varimax rotation, was used to examine the factor structure of each scale [28]. All analyses were conducted using SPSS version 20.

## Results

A total of 6,186 questionnaires were distributed among the kindergartens, and 4,553 were returned (response rate = 73.6%). A total of 2,360 (52.0%) of the children who participated were boys. A total of 1,572 (34.5%), 1,453 (31.9%), 1,468 (32.2%), and 59 (1.3%) children who participated were at grades 1, 2, 3, and nursery, respectively. A total of 777 (17.1%), 2,400 (52.7%), and 1,369 (30.1%) of the children who participated have attended full day, half day (a.m. only), and half day (p.m. only) class schedules, respectively. A total of 4,270 (94.3%) of the participating children were born in Hong Kong, whereas 257 (5.6%) were born in China or other places. A total of 4,328 (96.3%) questionnaires were filled by parents of the participating children. A total of 4,101 (93.3%) fathers had full-time or part-time job, whereas only 2,068 (47.2%) mother had full-time or part-time job. A total of 1,700 (37.7%) of the participating children were regarded as an only child, whereas 2,069 (45.9%) and 743 (16.5%) had one and two or more siblings, respectively. Other demographic information of the children and their parents were summarized in Table 1. The mean scores (standard deviations) for emotional feeding, instrumental feeding, prompting or encouragement to eat, and control over Eating, were 2.68 (0.66), 2.31 (0.70), 3.67 (0.64), and 3.82 (0.50), respectively.

**Table 1:** **Characteristics of the 4553 children and their parents**

Characteristic	^*^^$^Frequency (%)
*Children*	
Gender	
● Male	2360 (52.0%)
● Female	2175 (48.0%)
Grade	
● Nursery	59 (1.3%)
● Kindergarten 1	1572 (34.5%)
● Kindergarten 2	1453 (31.9%)
● Kindergarten 3	1468 (32.2%)
School status	
● Whole day	777 (17.1%)
● Half-day (a.m. only)	2400 (52.8%)
● Half-day (p.m. only)	1369 (30.1%)
Place of birth	
● Hong Kong	4270 (94.3%)
● China	210 (4.6%)
● Other place	47 (1.0%)
*Parents*	
Father’s job status	
● Full-time	3823 (87.0%)
● Part-time	278 (6.3%)
● Unemployed	129 (2.9%)
● Retired	27 (0.6%)
● Not applicable/ Unknown	136 (3.1%)
Mother’s job status	
● Full-time	1558 (35.6%)
● Part-time	510 (11.6%)
● Unemployed	2223 (50.7%)
● Retired	9 (0.2%)
● Not applicable/Unknown	82 (1.9%)
Father’s education level	
● Primary school or below	304 (6.8%)
● Secondary school	3092 (69.5%)
● Diploma/Degree or higher	963 (21.6%)
● Not applicable/Unknown	90 (2.0%)
Mother’s education level	
● Primary school or below	378 (8.4%)
● Secondary school	3272 (72.9%)
● Diploma/Degree or higher	790 (17.6%)
● Not applicable/unknown	50 (1.1%)
Relationship to the child	
● Father	580 (12.9%)
● Mother	3748 (83.4%)
● Others	165 (3.7%)
Siblings	
● 0	1700 (37.7%)
● 1	2069 (45.9%)
● 2 or more	743 (16.5%)

^*^Due to rounding error, the sum of percentage may not equal to 100%.

^$^Due to missing data, the sum of frequency may not equal to 4553.

### Internal reliability

The Cronbach’s alphas for emotional feeding, instrumental feeding, prompting or encouragement to eat, and control over eating were 0.81, 0.63, 0.83, and 0.63, respectively. Ranges of the item-deleted Cronbach’s alphas are shown in Table 2. The overall Cronbach’s alpha for all 27 items was 0.75.

**Table 2:** **Internal reliability of the parental feeding style questionnaire**

Parental feeding style questionnaire	Mean score (SD)	Cronbach’s alpha	Item-deleted Cronbach’s alpha
Instrumental feeding (4 items)	2.68 (0.66)	0.63	0.50-0.61
Emotional feeding (5 items)	2.31 (0.70)	0.81	0.74-0.80
Prompting or encouragement to eat (8 items)	3.67 (0.64)	0.83	0.80-0.82
Control over eating (10 items)	3.82 (0.50)	0.63	0.58-0.65
Total (27 items)	3.33 (0.36)	0.75	0.73-0.77

### Factor structure of the four scales

Factor analysis revealed a single factor for the four items of instrumental feeding which accounted for 47.72% of variance (eigenvalue = 1.91). A single factor structure was also revealed for the five items of emotional feeding, in which the factor explained 56.74% of variance with eigenvalue = 5.84. The factor loadings are shown in Table 3.

**Table 3:** **Exploratory factor analysis by scales**

	Factor 1$	Factor 2
*Instrumental feeding*		
In order to get my child to behave him/herself I promise him/her something to eat.	**0.77**	
我會為了讓孩子乖一點而應承給他一點吃的東西。		
If my child misbehaves I withhold his/her favourite food.	**0.66**	
如果孩子不乖或行為不端,我會拒絕給他所吃喜歡的食物。		
I use puddings as a bribe to get my child to eat his/her main course.	**0.60**	
我會用甜點來哄孩子,好使他好好地吃自己的主餐。		
I reward my child with something to eat when s/he is well behaved.	**0.73**	
當孩子乖和行為良好時,我會獎勵他一點吃的東西。		
*Emotional feeding*		
I give my child something to eat to make him/her feel better when s/he is feeling upset.	**0.71**	
當孩子不開心的時候,我會給他一點吃的東西來讓他感覺好一點。		
I give my child something to eat to make him/her feel better when s/he has been hurt.	**0.78**	
當孩子不小心弄傷時,我會給他一點吃的東西來讓他感覺好一點。		
I give my child something to eat if s/he is feeling bored.	**0.68**	
如果孩子覺得無聊,我會給他一點吃的東西。		
I give my child something to eat to make him/her feel better when s/he is worried.	**0.83**	
當孩子不安的時候,我會給他一點吃的東西來讓他感覺好一點。		
I give my child something to eat to make him/her feel better when s/he is feeling angry.	**0.76**	
當孩子覺得生氣的時候,我會給他一點吃的東西來讓他感覺好一點。		
*Promoting and encouragement to eat*		
I encourage my child to look forward to the meal.	**0.67**	
我會鼓勵孩子高興地期待用餐。		
I praise my child if s/he eats what I give him/her.	**0.67**	
當孩子吃我所給他的食物時,我會稱讚他。		
I encourage my child to eat a wide variety of foods.	**0.66**	
我會鼓勵孩子吃各種各樣的食物。		
I present food in an attractive way to my child.	**0.60**	
我會花心思向孩子呈現食物吸引的一面。		
I encourage my child to taste each of the foods I serve at mealtimes.	**0.70**	
在用餐時,我會鼓勵孩子試嚐所端上的每種食物。		
I encourage my child to try foods that s/he hasn’t tasted before.	**0.64**	
我會鼓勵孩子嘗試吃一些他從來沒有吃過的食物。		
I encourage my child to enjoy his/her food.	**0.74**	
我會鼓勵孩子享受進食的樂趣。		
I praise my child if s/he eats a new food.	**0.70**	
如果孩子吃一種新的食物,我會稱讚他。		
*Control over eating*		
I decide when it is time for my child to have a snack	**0.73**	-0.06
我會決定何時是時候讓孩子吃一些小食。		
I decide how many snacks my child should have	**0.73**	-0.01
我會決定孩子應該吃多少小食。		
I decide what my child eats between meals	**0.73**	0.03
我會決定孩子在餐與餐之間吃些甚麼。		
I decide the times when my child eats his/her meals	**0.66**	0.03
我會決定孩子在甚麼時候吃他的飯餐。		
I insist my child eats meals at the table	**0.47**	0.39
我堅持要孩子在餐桌用膳。		
I allow my child to choose which foods to have for meals*	-0.18	**0.46**
我會讓孩子選擇用膳時所吃的食物。		
I allow my child to wander around during a meal*	0.07	**0.61**
我容讓孩子在用餐期間四處閒逛。		
I allow my child to decide when s/he has had enough snacks to eat*	0.07	**0.65**
我會容讓孩子自己決定在甚麼情况下為之吃夠小食。		
I let my child eat between meals whenever s/he wants*	0.03	**0.65**
只要孩子想,我會讓孩子在餐與餐之間隨時吃東西。		
I let my child decide when s/he would like to have her meal*	0.08	**0.65**
我會讓孩子決定他想在甚麼時候用餐。		

*Reversed questions.

$: Bold numbers belong to the same factor.

A two-factor structure was initially revealed for the eight items of promoting and encouragement to eat, but the eigenvalues of the two factors were 3.63 and 1.03, respectively. After examining the scree plot, a one-factor structure was more appropriate, and the factor explained 45.36% of variance.

As to control over eating, a three-factor structure was initially revealed, and the eigenvalues were 2.42, 1.87, and 1.13. However, from the scree plot, a more appropriate two-factor model was fitted. The two factors, each consisting five items and can be categorized as restrictive control (e.g., “I decide when it is time for my child to have a snack.”) [29] and permissive control (e.g., “I allow my child to decide when s/he has had enough snacks to eat.”), [12] totally explained 42.90% of variance.

## Discussion

In this study, we translated the PFSQ into Chinese and revealed that the scale was fairly reliable, and the factor structure was consistent to that of other studies [23]. As far as we know, the current study was the first to translate and validate an established instrument on parental feeding style into Chinese although PFSQ was developed by Wardle and colleagues more than 10 years ago. We followed standard method to translate the instrument and pretested through some parents and hence the Chinese version PFSQ should be valid. In addition, a reasonably large sample was used in this study and the reliability analysis revealed an acceptable to good internal consistency for the overall and the four sub-scales. That implied the reliability of the Chinese version PFSQ.

A one-factor structure was found for emotional feeding, instrumental feeding, and prompting or encouragement to eat, whereas a two-factor structure was revealed for control over eating. The result is similar to the factor structure identified from the Turkish version; [23] however, one item “*I insist my child eats meals at the table*”, which was included as a restrictive factor in the current study, was also classified by Ozcetin as another factor; [23] hence, the construct validity is secured.

Having said that parents play a vital role for the healthy development of children, a valid and reliable tool for measuring the feeding style is indispensable. Our study did show that the Chinese version PFSQ is a proper instrument in this aspect and we expect the scale would be used in other Chinese-speaking population. Although there are a lot of positive results, there were still limitations of this study including (i) a non-random sampling which may limit generalizability, and (ii) test-retest was not performed which may limit reliability.

## Conclusion

The Chinese version of PFSQ has good reliability and validity in assessing parental feeding styles in Hong Kong. This instrument would enhance understanding on how parental feeding styles may affect the dietary patterns and ultimately the weight statuses of children. The validated instrument can also be conducted to compare parental feeding in Chinese population from different countries. In long-term, further exploration of the role of diet in early life may help prevent chronic non-communicable diseases.

## Authors’ information

Wilson Tam: Part of the work was conducted when the author worked at the Jockey Club School of Public Health and Primary Care, Chinese University of Hong Kong.

## Abbreviations

CFSQ: Caregiver’s Feeding Style Questionnaire
IFSQ: Infant Feeding Style Questionnaire
CFQ: Child Feeding Questionnaire
PFSQ: Parental feeding style questionnaire.
